# An Implementation Process Evaluation Based on an Integrated Psychosocial Support Program of Colorectal Cancer Couples in China: A Pilot Study

**DOI:** 10.3390/healthcare9020110

**Published:** 2021-01-21

**Authors:** Jieyu Li, Xingjuan Luo, Qiuping Li

**Affiliations:** Wuxi School of Medicine, Jiangnan University, Wuxi 214000, China; 6182806003@stu.jiangnan.edu.cn (J.L.); 6182806004@stu.jiangnan.edu.cn (X.L.)

**Keywords:** cancer, cancer couple, process evaluation, normalization process theory, psychosocial intervention, qualitative

## Abstract

Studies have shown that the qualitative process assessment of cancer couple-based psychosocial interventions is often ignored. This article aims to evaluate the implementation process of an integrated psychosocial program developed for colorectal cancer couples. Semi-structured qualitative interviews were conducted with eight colorectal cancer couple participants and two intervention facilitators. Normalization Process Theory was used to guide the data collection and analysis. Data analysis was conducted using a directed content analysis approach within a framework approach. Participants had a good understanding of the program significance. For most participants, the intervention duration was appropriate, and was well integrated into daily life. A lack of understanding of psychological nursing, and a lack of confidence in the use of online platforms and other personal factors, inhibited participants’ experience of participating in the intervention. The facilitator’s challenge in the implementation process was being flexible in dealing with situations occurring outside of the framework plan. Face-to-face and online psychological interventions require more flexibility, and participant cognition of psychosocial care was the key to the successful implementation of the intervention. Future research should consider raising participants’ awareness of psychological care to better integrate this type of intervention into participants’ daily lives and routine care.

## 1. Introduction

The diagnosis and treatment of cancer affects not only cancer patients, but also their family caregivers [1,2], particularly the spousal caregiver [3]. The impact of cancer and its treatment are comprehensive, including physical, psychological, social, economic, and other aspects [1]. The psychological effects are painful and profound. Psychosocial interventions for cancer date back to the 1970s and have been developed worldwide [4]. At the same time, studies have shown that cancer affects couples as a unit [5]. There are also a growing number of psychosocial interventions targeting cancer patient-spousal caregiver dyads [6,7,8].

Traditional psychosocial intervention for cancer patient-spouse caregiver dyads uses a face-to-face delivery format, which promotes open communication for couples and improves quality of life for both patients and caregivers [9]. However, there are some limitations to this, such as a high loss rate due to the limitations of time and space [9], and shyness in asking personal questions [10]. To provide a close and convenient support intervention, the Internet seems to be an appropriate option. Studies have shown the prominent advantages of web-based interventions, e.g., freedom from space-time as well as its anonymous nature [11,12,13,14]. A previous literature review of dyadic web-based interventions for cancer-patient caregiver dyads also supports the application of an Internet approach in achieving small to large positive effects across physical, emotional, and relational health aspects [15]. Therefore, we have developed a complex supportive program for colorectal cancer (CRC) patient-spouse caregivers that combines an online platform with face-to-face sessions: An integrated psychosocial support program for CRC couples. Intervention details have been published elsewhere [16].

On paper, psychosocial interventions may seem easy to implement. However, they can be difficult to implement in the real world [17]. For example, the fidelity of the intervention during transmission, the grasp of the intervention “dose”, and the influence of the facilitator’s (defined as the intervener who carries out the intervention) personal characteristics on transmission [18], all play a role. Other studies have shown that psychosocial interventions are more difficult to implement than drug or surgical interventions [17,19]. This is one reason that psychosocial interventions are called complex interventions [19]. It is also proposed that it is difficult to know why complex psychosocial interventions work without exploring their underlying processes [17,20,21].

Process evaluation involves explaining the reasons for the success and/or failure of the intervention experiment and how to optimize it, evaluating the fidelity of execution, and exploring and identifying contextual factors associated with the outcomes [19,22,23,24,25,26]. It is more useful to understand how the end results are achieved and what factors contribute to or inhibit the end results, than simply presenting the final outcome in terms of numbers and statistics. Process evaluation is widely used in both randomized controlled trials and feasibility studies [21,27]. It is particularly important for complex psychosocial intervention trials, and can explore participants’ views and modify them before a larger trial is undertaken [17,20]. A recent literature review of qualitative evaluation in nursing interventions supports the view that “continuous evaluation during the implementation process is crucial for success” [28], p. 1296.

Consequently, in parallel with the feasibility study on the integrated psychosocial support program developed for CRC couples, the current study was designed to conduct a process evaluation. The specific purposes of the process evaluation were to understand the overall intervention process, to explore factors that promote and/or hinder program implementation, and to provide evidence for further modifying the program before conducting large-scale trials.

## 2. Methods

### 2.1. The Intervention

Briefly, our intervention included six sessions. The specific flow diagram of the intervention is shown in Table 1. The duration of the online study is from weeks 1 to 5, and the face-to-face sessions are weeks 2, 4, and 6. The first week also includes the baseline survey and the introduction to the online platform. The online learning sessions mainly include psychoeducation, information support, skills-building, and online communication support. The face-to-face sessions were designed to reinforce the online learning content. In addition, participants can access resources and study at any time through the online platform, whether the course is in progress or not. The main outcome measurements for the effect evaluation include recruitment rate, completion rate, self-efficacy, dyadic coping, cancer-related communication, quality of life, and positive (benefit finding) and negative (anxiety and depression) emotions. The program feasibility study was conducted from October 2019 to January 2020, with the intention of examining the feasibility, acceptability, and preliminary efficacy of the integrated psychosocial support program for CRC couples [16]. It is hypothesized that the program would promote CRC patients and their spousal caregivers to positively cope with cancer together and improve their quality of life.

### 2.2. Implementation Theoretical Framework

As a sociological theory, Normalization Process Theory (NPT) involves the social organization of work (implementation), making practice a regular element of daily life (embedding), and maintaining embedded practice in its social context (integration) [22,23,29]. NPT has been widely used to assess the success or failure of, and understand the dynamics of, complex intervention practices [29,30]. Murray et al. also suggested that researchers should consider whether interventions can be widely implemented and integrated into daily life before larger studies are conducted [25]. Therefore, we chose NPT as the theoretical framework to guide the current process evaluation in data collection and analysis.

### 2.3. Evaluation Design

A qualitative approach of semi-structured face-to-face interviews was applied to explore participants’ experiences, perceptions, and issues related to the intervention implementation. A semi-structured interview is an informal interview conducted according to a broad-line interview outline (only basic requirements are proposed and core questions are drawn up). The specific interview questions are adjusted in time according to the actual situation during the interview, to achieve effective communication with the interviewees and obtain comprehensive and sufficient first-hand information [31]. At the same time, face-to-face interviews are more conducive to visual transmission and observation of emotional expression [32]. This approach is considered appropriate when exploring the feelings and reasons directly related to the underlying process of intervention [32,33].

### 2.4. Participants and Data Collection

We invited CRC patients and spousal caregivers who had participated in the program’s feasibility study to share their understanding and experiences of the project. Eligibility criteria were: adult married couples with one partner diagnosed with CRC (any stage); patient’s primary caregiver was his/her spouse; couples had daily access to a smartphone (some older cancer couples do not have smartphones, while some have smartphones but do not use them in their daily lives); and both patient and partner could communicate in Mandarin and were willing to participate in the program. The two facilitators were also interviewed. The interviews were conducted from January 2020 to October 2020.

Face-to-face semi-structured interviews with colorectal cancer patients and spousal caregivers were conducted by the first author (LJ) to explore participants’ real experiences and feelings in the process of practice. The semi-structured interview schedule developed under the NPT framework is shown in Table 2. All participation was voluntary. Participants were fully informed of the details on the study they were participating in and before participating, agreed verbally to be interviewed and recorded. Each couple was interviewed individually in a closed meeting room in the oncology department. Each interview lasted 30–60 min. LJ also conducted face-to-face semi-structured interviews with the two facilitators to explore their experiences throughout the intervention. In addition, we collected diaries from the facilitators.

At the end of each interview, LJ provided a brief overview of the main issues discussed, and participants had an opportunity to clarify their views. The recordings were transcribed verbatim at the end of each interview. All audio and text content will be available only to members of the research team.

### 2.5. Data Analysis

All interviews were transcribed verbatim. Based on the four structural framework components of NPT, we used directed content analysis methods within a Framework Approach [34] to analyze and encode the transcribed data. First, we carefully read and re-read the transcripts to deepen our familiarity with, and understanding of the data. Data were then encoded and broadly encoded into the four structures of the NPT. Following this, the data were encoded in more detail into specific components of each NPT structure. For example, data related to collective action were re-read and further encoded into content, outcome measures and other substructures. During this process, data would not be coerced into the NPT framework. One study showed that using NPT to guide interviews, collect data, and interpret results minimizes researchers’ biases [26]. In fact, the data analysis process began at the same time as the data collection. All data were analyzed independently by the first (LJ) and second (LX) authors. Any inconsistencies and conflicting interpretations were resolved through discussion. If they could not be resolved, a discussion was conducted by the research team to reach a consensus.

### 2.6. Ethical Considerations

This study was approved by the research ethics committee of Jiangnan University (JNU20200731RB01), and access approval was obtained from the Affiliated Hospital of Jiangnan University. All participants gave written informed consent before participating in this study. To protect participants’ privacy and anonymity, we replace participants’ names with special codes, for example, P1, P2 (for patients) … SC1, SC2 (for spousal caregivers) … F1 and F2 (for facilitators). All data were kept in a sealed data cabinet within the study group, accessible only to authorized personnel, and all data were destroyed at the end of the study.

## 3. Results

Table 3 shows the participant characteristics. Among the eight couples, the mean ages of the CRC patients and spousal caregivers were 58 years and 60 years respectively. Half of the CRC patients and spousal caregivers were male. The mean length of their marriage was 34 years. All CRC patients had advanced cancer, with one year in average time since diagnosis. One facilitator is a graduate student who studied interviewing for two years and has nine years of clinical work experience. Another facilitator is a senior nurse with 20 years’ experience of clinical work. Based on the four theoretical structures of the NPT, the findings of this process evaluation are described and explained using the following four theoretical structures: (1) coherence—the sense-making work: understanding the purpose, meaning, feasibility, and necessity of the intervention; (2) cognitive participation—the relational work: exploring related factors that promote and/or inhibit participants from engaging in the program; (3) collective action—the enactment work: understanding the ways that participants interact with the intervention to make them work, and the promotion, hindrance, and challenges encountered in the actual intervention process; and (4) reflexive monitoring—the appraisal work: conducting an evaluation of the effects and providing recommendations for further intervention improvements.

### 3.1. Coherence—The Sense-Making Work

In this section, we report participants’ overall understanding of our intervention program, and how they understood the intervention purpose. Featherstone et al. reported that participant understanding of an intervention project influenced the outcome of the trial [35].

Most participants had a good understanding of the meaning of the intervention and expressed their agreement with the intervention purpose:

*I have to admit that I did not fully understand the meaning of the intervention before I participated in it… There is no doubt that it takes practical application to understand and experience the wonders of psychological care. (SC6)*

Participants understood the potential benefits and expected the project to have a positive impact on their lives. For example, some participants reported that they expected that participating in the program would improve their quality of life, as they were gaining information about how to standardize dietary care and reduce anxiety:

*I’ve always thought that the oncology ward should be staffed by a psychiatrist...But I feel like it’s going to be very difficult right now...After reading your recruitment information, I would like to participate in it…because that is really what I need. (P3)*

Participants also understood the significance of associating face-to-face sessions with an online platform, believing that this novel approach brought them a great deal of convenience:

*The emergence of online platforms has really helped us a lot, making up for the lack of face-to-face interactions… During the COVID-19 epidemic, we were also unable to go to the hospital, which made us very anxious… Fortunately, there were online platforms, regular updates, and online consultations. (SC8)*

Some participants expressed their appreciation for having an official and authoritative cancer-learning website to keep up with the latest cancer news, and specifically for colorectal cancer. In addition, they could ask questions online and receive timely answers:

*Before participating in your program, I usually accessed the Baidu engine and tried to search for the information that I needed. Unfortunately, where the information is mixed, I cannot distinguish what is right and what is wrong, so I’m always confused…Now I can ask questions on the online platform…I think it’s quite good…I think it will be very reliable and trustworthy. (P2)*

### 3.2. Cognitive Participation—The Relational Work

In this section, we mainly explore whether participants participate in and commit to using online platforms, and what factors promote and/or inhibit such commitment. At present, smartphones are widely used in China [36]. Meanwhile, as the most popular social online platform, WeChat provides a new way for the public to receive health interventions [37,38,39]. Participants thought it was convenient to learn lessons while using their mobile phones on a daily basis.

#### 3.2.1. Training and Support

Prior to the start of the intervention, participants attended a lecture and were trained in the use of the online platform. This mainly included an introduction outlining the project significance, purpose, final expected benefits, and face-to-face guidance in using the online platform. Participants also expressed appreciation for the combination of group and individual training:

*The format and time are more flexible… If I don’t understand anything after the lecture, I can ask for your support through the online platform, which is helpful and convenient. (P8)*

#### 3.2.2. Simple and Convenient

Almost all participants reported no financial worries, everyone had a smartphone and did not need to purchase a computer. At the same time, few participants reported the need to learn new skills to use the online platform. It was generally believed that the online platform was easy to operate, with a clear interface design, and it was easy to find the desired information. They also appreciated the anonymity, which protects personal privacy. People with a limited ability to use the Internet also reported that they felt confident and comfortable during the face-to-face classes.

#### 3.2.3. Facilitator’s Identity

The participation or presence of familiar senior managers can be a significant factor in encouraging participants to enter the intervention, and can increase their enthusiasm for participation:

*In the initial stage, it is very helpful that a senior manager or head nurse reintroduces the facilitator’s identity and integrated projects to the participants, which can reduce a participant’s vigilant psychology, raise the participant’s trust in both the facilitator and the program, and promote a participant’s entering into the intervention… It feels like we will soon be “inside them”. I think that the nurse was just giving a brief introduction, but it was a great motivator indeed… Patients and caregivers have a high level of trust in the role of the nurse… It’s the subtle relationship between the nurse and the patient. (F1)*

#### 3.2.4. Barriers to Engaging with the Program

Some participants felt that the psychological intervention did not seem to have much effect and did not offer anything novel:

*I don’t think it’s going to provide me with any benefit, and it won’t help me with my disease... These things only change one aspect… Nothing else has changed. It doesn’t really appeal to me. (P6)*

Participants believed they did not need help, their miserable experiences were incomprehensible to others, and that others were even “consuming” them:

*All I care about every day is my medication…I’m fine now… I don’t … And others will not understand my pain…How can anyone else understand when they haven’t experienced it? (P5)*

This participant thought his emotions were self-regulating, and he did not want to bother others:

*He doesn’t like to express his emotions, and gets used to keeping them inside...For example, he seldom calls me when he needs help. Even during the hospitalization, for fear of troubling the medical staff, he rarely consulted doctors and/or nurses. (SC8)*

Some participants were less confident in their ability to use a smartphone and less confident in learning through an online platform:

*Although I use WeChat every day, I always forget to click into the official account to learn…I don’t feel like I have enough flexibility with my smartphone yet. (SC3)*

### 3.3. Collective Action—The Enactment Work

Another purpose of NPT was to explore the facilitating and impeding factors embedded and integrated into the actual intervention process, while the focus of psychological intervention support was also to explore the degree of implementation in real life. Psychological intervention support may work well on paper, but is often difficult to implement in practice [17,19]. Most participants completed six sessions, and all participants completed the face-to-face sessions. The intervention duration was six weeks, which was acceptable to most participants.

#### 3.3.1. Online Platform Login

A few participants encountered problems during the login process. For example, they needed to log in again when they had not used the public account for several days, as they did not receive the verification code when they logged in. In these cases, the participant had to determine the reason for the login failure and log back in, creating a little additional work for themselves. Nevertheless, participants believed that it was easier for them to use a smartphone than a computer:

*Once I forgot to enter the online platform for a few days…When I entered it again, I was required to enter the verification code. Maybe there was something wrong with my mobile phone, because I didn’t receive the verification code until then.…Even so, I think it’s easier than using the computer. (P4)*

#### 3.3.2. Integrated Intervention Content

Most participants felt that the content was comprehensive, and although the intervention’s main focus was psychological knowledge, it also provided other forms of support, such as information about disease, diet and nutrition. However, some participants would read all of the psychological knowledge at once, and then they would not access the online platform if there was no updated information. The majority of participants found the psychological knowledge descriptions easy to understand. In the face-to-face sessions, participants preferred that facilitators shared positive real-life cases of relevant aggressive cancer treatments:

*Sometimes, I find that the online platform knowledge includes too much text, which may interfere with my interest in reading and makes me feel bored…Is there an alternative form, like picture dialogue? … I have noticed some positive examples in the updates, but I feel it is too far away from me…it feels too unreal. (P1)*

#### 3.3.3. Measurement of Results

There are different opinions on the measurement of the results. Some people said that these measurements were comprehensive and made them rethink what cancer had given them. Others thought that the task of measuring results at the two time points was very tedious. For example, in the questionnaire for patients and spouses, there were both positive and negative questions, and they needed to spend considerable time and energy filling out the questionnaire. In addition, perhaps for personal reasons, some participants did not want to answer personal questions, such as questions about sex, in the questionnaire:

*…That’s a lot of questions...Why are these questions (sex problems) being asked here? It’s awkward. (P2)*

#### 3.3.4. Problems Encountered by Facilitators in Implementing Intervention

Participants were more likely to talk and confide in the facilitator during the first session, making it difficult for the facilitator to intervene. For example, the facilitator would not interrupt the participants when they were talking about their traumatic experiences, leading to the extension of the intervention time:

*…The participants were sad, and some even cried, as they talked about their experiences… At this point, we should not interrupt their conversation, but listen silently and respond with a little… I remember that one time, after communicating with a patient for over an hour, he asked a lot of questions about nutrition and medical reimbursement, which he was eager to address… I couldn’t interrupt him. I think that they may need someone to talk to, and I hope that our intervention will help them to feel comforted. (F2)*

One challenge for facilitators was that the six sessions of the intervention were always difficult to implement as planned, with participants always asking new questions that needed to be addressed at that time. Therefore, facilitators did not have enough time to handle what already needed to be dealt with in each session:

*To complete each intervention on time, we always started the course early. Because it takes time to deal with new problems. (F1)*

Whether in a face-to-face or online course, facilitators must choose the right educational moment. This is because participants were not always open during the intervention, due to a variety of reasons, including being tired, being in therapy, and being particularly depressed and unwilling to communicate at certain times:

*Even if the participant was willing to participate in the intervention, we have to find the appropriate time to deliver the intervention… We choose to deliver when they are comfortable and energetic. (F1)*

Because interventions were targeted at the patient and spousal caregiver as a unit, the implementation process was often hindered by problems experienced by one of the partners:

*Sometimes, one of the partners, e.g., husband or wife, can’t come with their spouse because of some reason, so we have to adjust our implementation strategy and be flexible. (F2)*

*Some patients did not want to know everything about the disease, such as treatment and medication, diet and nutrition. Patients may feel overwhelmed if they receive too much information, and they just want to listen to their doctors for their treatment. So patients are less motivated to participate in the intervention than spousal caregivers. (F1)*

In practice, the implementation process actually needs a long period of time, and is a gradual process.

*We need to start from the unfamiliar to the familiar, we need to gradually gain trust and understanding...In fact, after getting to know them and meeting with them a few times, they are willing to open up to us, slowly start talking, and are willing to share their true emotions and current difficulties. (F2)*

#### 3.3.5. General Overall Obstacles

Participants’ verbal expressions were inconsistent with their actual actions; they were always trying to be brave, so they were always in conflict, sometimes thought they needed help, and sometimes thought they were doing well and did not need help:

*I hope you can come and chat with him often...He always reads all kinds of articles with positive energy and told me that he would be positive. But in fact, he could not actively face and comply with the treatment when he was actually facing the treatment, and often had bad and terrible ideas. (SC7)*

*When I communicated with the patient, he kept telling me, “I’m very positive now”, and one patient even showed me the positive articles posted on his social account. However, from the conversation process and the description of the caregivers, it could be understood that the patient’s verbal and actual emotions were not consistent. (F1)*

During the process, participants were reminded of their past cancer experiences, which would cause sadness, and they were also constantly reminded that they were a cancer patient:

*I would always look back on the countless chemotherapies from the diagnosis to the countless times that followed, and I would feel guilty about my husband, for all he had done for me...Sometimes, I would try to distract myself from everything to do with the cancer, but the more I struggled with it, the less I could escape it. (P4)*

### 3.4. Reflexive Monitoring—The Appraisal Work

Post-intervention feedback was also important for the process evaluation. It mainly includes evaluating the effectiveness and proposing reasonable improvement suggestions for future large-scale implementation. The facilitators suggested that comprehensive psychosocial intervention support was more effective for those who wanted to actively face the disease, but did not find an appropriate approach.

#### 3.4.1. Benefits and Changes

Most participants were able to identify the positive impact of their participation. Many couples improved their communication through the intervention:

*I think that either participant face-to-face sessions or learning through online resources on our own allows us to positively face our problems as a couple…I think I communicated with my husband a little bit more after taking part in the program, because he would always remind me to remember what we were taught in the course. For example, instead of being silent, we should talk to each other. (P3)*

Post-implementation, a number of participants were aware of the importance of a couple’s psychological state during cancer treatment:

*Before taking part in the program, I rarely mentioned anything about her illness in front of her, for fear of upsetting her. However, as time goes by, both sides will find this to be an uncomfortable state, due to the lack of sharing our experiences with each other…In the process of learning through the online resources and communicating with you, I found that my state of mind was getting much brighter, and my communication with her also changed a lot…I think the psychological support is quite useful, and I will continue to pay attention to it in the future. (SC2)*

Some participants reported that they had the opportunity to talk and find a release, giving them a temporary escape from the treatment for their illness:

*In fact, in the process of participating in the study, I focused more on the conversations, talking to you about what I was afraid to tell my wife and children, which made me feel relaxed. (P1)*

After the implementation, some participants became more positive and more involved in their treatment:

*I think he has changed a lot after going back home…He would often ask questions on the online platform, such as what would be more nutritious to eat? Should I eat this fruit more? It feels like he’s less pessimistic...I was relieved that he didn’t give up on himself because every time I went out to work, he would be at home alone, and he would be able to take good care of himself. (SC1)*

Several participants benefited from the process rather than from the online or face-to-face sessions. For example, some people felt that someone was beginning to pay attention to their mental state, and they were not just going through the motions in therapy. Others described the intervention as giving them a sense of anticipation and motivation:

*Honestly, I looked forward to your arrival, which makes me feel like I have something to do while staying in the hospital…every time you come and talk to us, particularly to my wife, no matter what you have talked about, she would feel much better than before. (SC4)*

#### 3.4.2. Online vs. Face-To-Face

Most participants thought that the delivery form was flexible, that they could go to face-to-face meetings with the facilitator during their spare time while they were in hospital for chemotherapy, and that they could learn by taking courses on the online platform at home. Some couples realized their previous misperceptions and discussed them while using the online platform. Some couples did not communicate with each other while using the online platform, but the changes in their actions could be seen in later face-to-face classes. As for the delivery method, some participants expressed their preference for a face-to-face delivery approach. This may have been due to the following reasons: they seldom use a smartphone, they have a weak awareness of online learning, and they prefer to receive information by talking with others. As one participant explained:

*I like to get feedback from people via face-to-face communication, which makes me feel like someone is responding to me…And I often forget web learning. (SC5)*

Many participants rated the function of asking questions in the online platform highly, sometimes valuing this even more highly than the course setting. The reasons for participants’ interest in online learning were: frequent attention to the dynamics of the online platform, the convenience of asking online questions and the timely answers, freedom from time and space restrictions, and the protection conferred by anonymity and privacy. Participants preferred the online platform’s convenience and flexibility, without the need to interrupt their plans to take extra time out to participate:

*Every day when I pick up my phone, the first thing that I pay attention to is the news. Although I am old, I can still make videos… Am I very fashionable (laugh)? Sometimes I lost the paper materials that you gave me during the face-to-face intervention, and I think it is quite convenient to learn from the online platform... (P2)*

#### 3.4.3. Full Participation and Seamless Integration

Some participants wanted to be involved from cancer diagnosis through treatment, and not just at one of the disease stages:

*When I was first informed about his diagnosis, I couldn’t accept it. To me, he had never been ill and he kept exercising...I didn’t dare tell him the truth. At that time, I really wanted someone to help me...After several treatments, he noticed that something was wrong and asked me: Was it a tumor? Slowly he and I came to terms with the fact...I think it’s good to let nature take its course...Now we are getting better... If only we had been a part of this project from the beginning. (SC8)*

#### 3.4.4. Improving the Intervention

The suggestions put forward by the participants were more about the function of the online platform. Participants mentioned that they would often forget to enter the course of the official account, and hoped that a convenient reminder function could be set up. The updates should be more frequent, so they would continually have new knowledge to learn. Other participants suggested reducing the amount of text and adding more pictures, to make it more intuitive. Another concern was that, although the delivery was a combination of online and face-to-face approaches, the chances of learning in an online course would be greatly reduced for those with weak smartphone skills.

## 4. Discussion

The process evaluation describes the facilitating and impeding factors in the intervention process, clearly conveys the overall intervention process, and provides information for the implementation of future interventions. Within the NPT’s four-part structural framework, participants were better able to identify problems encountered in the participation process and provide improvement recommendations to facilitators.

In coherence and cognitive participation, we found that participants’ understanding of the importance of psychological care in the overall treatment of the disease may be the key to successfully implementing psychological interventions. In addition, the participation completion rate can also be increased. A qualitative study found similar results, particularly in terms of the importance of participants’ understanding of the project [40]. In fact, in China, cancer patients are not very good at expressing their emotions, especially negative emotions [41]. What is more, cancer patients and spousal caregivers often do not disclose negative emotions to one another, because of their intention to protect the other person [2,42]. Cancer patients and caregivers do not understand the benefits of open communication and do not pay attention to one another’s communication needs [43]. Therefore, more effort should be put into participant recruitment, to reach more people and improve their understanding of the value of psychosocial intervention.

In collective action, the seamless integration of the implementation process into participants’ daily lives and therapeutic care is an important factor in promoting the successful implementation of the project in clinical workflow. For example, some participants lacked the confidence to use the online platform in their daily lives, and the perception that using an online platform was “extra work” also hindered, to a certain degree, by the intervention implementation. Similar results were found in a qualitative process evaluation study [44]. Participants focused on the fact they could naturally learn about psychological care while undergoing treatment, without having to expend additional energy.

In the psychological intervention process, the facilitators also encountered many challenges. Our study highlighted that the implementation of psychosocial interventions requires the facilitator to be flexible in dealing with a variety of unforeseen circumstances, which is also a great test for facilitators. At the same time, it is important to ensure the implementation fidelity of complex psychosocial interventions. Similar results were found in the process evaluation of other psychological interventions [17]. Several studies have reported that the facilitator’s personal characteristics [18,45], as well as attitudes and beliefs about the intervention [17,46], influence psychosocial intervention delivery. Conversely, one study has suggested there is no difference in psychological intervention delivery, whether by novice or experienced facilitators [47]. Therefore, future research is required to identify the facilitator’s role in the implementation of psychosocial interventions.

In reflexive monitoring, findings of improved communication between couples may be due to the program features that foster patient-spousal caregiver sharing, and offer online communication support. This is consistent with the findings of a web-based support program for prostate cancer patients and their caregivers [48]. Almost all participants were satisfied with the combination of face-to-face and online delivery because of the flexibility. Participants can learn using an online platform, without having to acquire new skills or buy new equipment. Online platforms have become particularly important during the COVID-19 pandemic. Cancer patients and caregivers face a double stressor during the emergency period. On the one hand, treatment of cancer patients is delayed due to hospital restrictions on patients and caregivers [49]. On the other hand, cancer patients are more vulnerable to the virus because of their low immune function [50,51]. So they have been forced to wait anxiously in isolation at home. At this point, timeliness and anonymity were highly valued by participants. One of the great advantages of being online is that participants can ask questions and receive answers quickly. More importantly, the privacy concerns of colorectal cancer patients can be addressed by asking questions online. In fact, during the process evaluation, some of the older participants were also found to be proficient with smartphones. This result has been confirmed in previous studies [52,53]. There is also more and more research devoted to the development of mobile phone functions or apps suitable for older adults [54,55]. When choosing face-to-face courses, participants can share their feelings and thoughts with facilitators during the learning process. In short, whether the delivery of psychosocial support is in person or online, it is important to maintain the flexibility of the intervention, which helps ensure intervention fidelity. In addition, a systematic review shows that in evaluating web-based interventions, attention should be paid not only to usability in terms of web design, but also to participants’ psychological experiences [56].

Although web-based psychological intervention is becoming increasingly popular, face-to-face intervention is still indispensable and irreplaceable, because in the process of psychological intervention transmission, what is important is that the facilitator responds to participants’ emotions and maintains empathy throughout the process [17,18,57]. This is missing from the other approaches. Therefore, the best way to deliver the intervention may be a combination of face-to-face and online approaches.

### 4.1. Study Limitations

It must be admitted there are some limitations in this study. Interviewing the couples at the same time may have influenced them to say things they thought the other person wanted to hear. Future large-scale studies should consider interviewing patients and spousal caregivers separately. One of the challenges in interviewing patients and caregivers separately is increasing the interviewer workload. In addition, when interviewing patients and caregivers separately, it is not possible to observe couples’ interactions. Another limitation is that the interviewers were also involved in the feasibility study implementation and may have “guided” the development to some extent. In addition, although it is a qualitative study, our study’s small sample size may limit its applicability and the impact of the achieved results. It would be safe to consider this as a pilot study. A further expanded sample size in future studies is highly recommended.

### 4.2. Implications for Practice

Although there are some limitations in this study, the results also have a number of implications for practice. Clinical health workers should do more to publicize the importance of psychological care to cancer patients and caregivers, and enhance their awareness of psychological care. In clinical practice, psychological nursing should be relatively seamless in the lives of patients and caregivers alike, so that psychological nursing becomes a part of routine clinical nursing. At the same time, both clinical workers and facilitators should maintain empathy and consider the perspectives of both patients and caregivers. What is more, facilitators should be trained in more diverse scenarios, to enhance their ability to respond flexibly and to ensure that all intervention objectives are implemented.

## 5. Conclusions

The process evaluation study identified several contributing and inhibiting factors. Contributing factors include flexibility, in the form of intervention delivery without the need to learn new skills or buy new equipment. A lack of understanding of psychological nursing, and lack of confidence in the use of online platforms and other personal factors, inhibited participants’ experience of participating in the intervention. The facilitator also plays an important role in the implementation of any psychosocial intervention. Increasing participants’ awareness of the importance of psychosocial interventions is the key to successful intervention implementation. Future research should consider better integrating interventions into participants’ daily lives and routine care, while raising participant awareness of psychological care, to help ensure successful implementation in larger trials.

## Figures and Tables

**Table 1:** The flow diagram of intervention.

Face-To-Face Sessions		FTF 1 Review Online Session 1 and Session 2		FTF 2 Review Online Session 3 and Session 4		FTF 3 Review Online Session 5 and the Whole Program
Time point		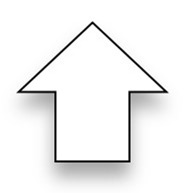		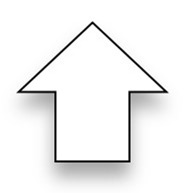		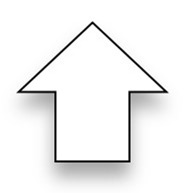
	**Week 1** (baseline surveys)	**Week 2**	**Week 3**	**Week 4**	**Week 5**	**Week 6** (post-treatment surveys)
	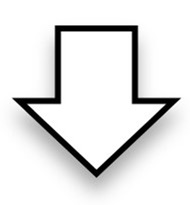	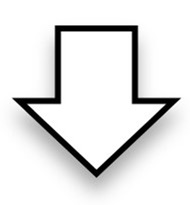	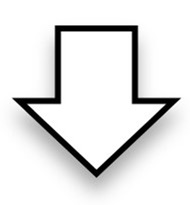	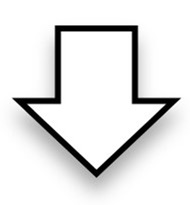	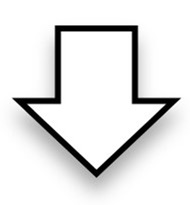	
Online sessions	**Session 1** Take care of your spouse with cancer	**Session 2** Adapt to the role as patient/caregiver	**Session 3** Mutual support and coping together	**Session 4** Effective and genuine communication	**Session 5** Rebuild confidence and return to society	

Abbreviations: FTF1: face-to-face session 1; FTF2: face-to-face session 2; FTF3: face-to-face session 3.

**Table 2:** Guiding questions for each interview for participants and facilitators.

**For Participants:**
1. Can you tell me your understanding of this project? Did you think it makes sense? Do you feel like you’ve changed since you joined the program?
2. Can you tell me how you were involved in the study? What motivated you to participate in this study? Why did you decide to become involved?
3. Did you have any problems participating in the study? Or was there any difficulty in using the online platform? We would like to know about your experience of using the online platform.
4. What did you think of the face-to-face and online platforms?
5. Could you tell me what your overall experience was?
6. Did you have any suggestions on how to improve our study? Or suggestions for an online platform?
Additional relevant questions were asked in response to the participants’ dialogues.
**For Facilitators:**
1. What did you think were the conveniences and obstacles in the implementation process?
2. Where did you think the research needs to be improved?

**Table 3:** Patient and spousal caregiver characteristics.

		Age(y)	Gender	Marriage Length (y)	Education Level	Informed about the Disease	Cancer Stage	Time Since Diagnosis (y)	Length of Time as a SC	Type of Treatment	Stoma	Health Status
P	P1	64	male	40	Middle school	well informed	A	1.8		chemotherapy	Yes	as usual: feel normal
	P2	60	female	36	Middle school	partly informed	A	0.3		surgery	Yes	as usual: feel normal
	P3	45	female	25	Middle school	well informed	A	1		chemotherapy	Yes	as usual: feel normal
	P4	55	female	35	Middle school	well informed	A	0.6		chemotherapy	No	as usual: feel normal
	P5	66	male	42	Primary school	partly informed	A	1.2		Radiotherapy + chemotherapy	No	poor: does not feel well
	P6	46	female	20	Undergraduate education	well informed	A	0.6		Chemotherapy + surgery	No	as usual: feel normal
	P7	56	male	31	Undergraduate education	well informed	A	0.7		Chemotherapy + surgery	Yes	good: feel well
	P8	72	male	42	Primary school	partly informed	A	1.8		Chemotherapy + surgery	Yes	as usual: feel normal
SC	SC1	64	female	40	Primary school	well informed			6~24 months			as usual: feel normal
	SC2	61	male	36	Primary school	partly informed			<6 months			as usual: feel normal
	SC3	48	male	25	Middle school	well informed			6~24 months			good: feel well
	SC4	61	male	35	Middle school	partly informed			<6 months			as usual: feel normal
	SC5	70	female	42	Primary school	partly informed			6~24 months			as usual: feel normal
	SC6	45	male	20	Undergraduate education	well informed			<6 months			good: feel well
	SC7	56	female	31	Middle school	well informed			6~24 months			as usual: feel normal
	SC8	71	female	42	Primary school	partly informed			6~24 months			poor: does not feel well

Abbreviations: P, patient; SC, spousal caregiver; y, year; A, advanced stage: represents distant metastasis in the present sample.

## Data Availability

The data presented in this study are available on request from the corresponding author. The data are not publicly available due to Participant’s personal information.
